# Development of an LC-MS/MS method for quantification of colistin and colistin methanesulfonate in human plasma and its application to stability studies and therapeutic drug monitoring

**DOI:** 10.1016/j.jmsacl.2025.05.001

**Published:** 2025-06-01

**Authors:** Tinghui Zhao, Lu Liu, Guangjie Yang, Hengyi Yu, Lihui Qiu, Xiping Li, Dong Xiang, Xuepeng Gong

**Affiliations:** a Department of Pharmacy, Tongji Hospital, Tongji Medical College, Huazhong University of Science and Technology, Wuhan, Hubei 430030, China; b Department of Pharmacy, Wuhan Mental Health Center, Wuhan, Hubei 430030, China

**Keywords:** Colistin, Colistin methanesulfonate, Liquid chromatography-tandem mass spectrometry, Pharmacokinetics, Stability

## Abstract

**Introduction:**

Colistin serves as the last line of defense against multidrug-resistant Gram-negative bacterial infections and is commonly administered in clinical practice as its prodrug, colistin methanesulfonate (CMS). However, due to its notable nephrotoxicity and narrow therapeutic window, therapeutic drug monitoring (TDM) is essential.

**Objectives:**

To develop an optimal LC-MS/MS method for the quantification of colistin and CMS in human plasma and to apply it to stability studies and TDM.

**Methods:**

Colistin A, colistin B, and internal standard (IS, polymyxin B2) were extracted from plasma using solid phase extraction columns. Sample separation was performed using a Welch Ultimate LP-C18 column with a 5-minute gradient elution consisting of water and acetonitrile, both supplied with 1.0% formic acid. The CMS concentration was obtained by comparing the total amount of colistin in acid-hydrolyzed and non-acid-hydrolyzed plasma.

**Results:**

Colistin A and colistin B showed excellent linearity in the concentration range of 0.1–10.0 μg/mL (R^2^ > 0.995) with acceptable specificity, accuracy (90.97 %–114.65 %), precision (RSD < 15 %), matrix effect (RSD < 15 %), and recovery (91.93 %–100.93 %). CMS in five commonly used clinical infusion solutions was stable when stored at room temperature for 8 h or at 4 °C for 24 h. The whole blood and plasma samples of CMS are susceptible to degradation at room temperature but are stable on ice. Plasma concentrations of colistin and CMS were accurately determined in three critically ill patients.

**Conclusion:**

The method we have developed is robust and streamlined, and has successfully demonstrated the potential feasibility for future TDM applications of colistin and CMS in critically ill patients.

## Highlights

• Fully validated LC-MS/MS method for qualification of prodrug CMS and colistin.
• Rapid, simple, and inexpensive with wide linear range.
• CMS stability in infusion solutions, blood, and plasma was investigated.
• Method was successfully applied to a pharmacokinetic study and TDM.

## 1 Introduction

Polymyxins are peptide antibiotics produced by *Bacillus polymyxa*, which primarily exert their antibacterial effects by increasing the permeability of bacterial cell membranes [1]. The antibacterial spectrum of polymyxins mainly includes Gram-negative bacteria such as *Escherichia coli*, *Klebsiella species*, and *Enterobacter species*. Polymyxins are particularly effective against carbapenem-resistant Gram-negative bacteria, such as multidrug-resistant *Pseudomonas aeruginosa*, *Acinetobacter baumannii,* and *Klebsiella pneumoniae* [2]. However, due to the significant nephrotoxicity and neurotoxicity of polymyxins, they were withdrawn from the market after being developed in the 1950s. Nowadays, with the increasing prevalence of multidrug-resistant Gram-negative bacterial infections, polymyxins, as the last line of defense, have been re-used in critically ill patients [3]. Polymyxins have five main variants: polymyxin A, B, C, D, and E. Currently, polymyxin E, also known as colistin, is one of the most commonly used in clinical practice [4].

Colistin is a complex mixture composed of at least 30 components, with colistin A and colistin B being its main constituents. These two components typically account for more than 85 % of the total colistin [5]. Colistin is typically used clinically in the form of its prodrug, colistin methanesulfonate (CMS). After conversion into colistin in the body, CMS exerts antibacterial effects [6]. Colistin is highly nephrotoxic and has a narrow therapeutic window. The drug concentrations for antibacterial activity overlap highly with the concentrations that induce nephrotoxicity, with a high incidence of renal injury often observed in the clinical use of CMS [7,8]. Furthermore, low drug exposure may not only fail to combat infection but also increase the risk of drug resistance [9]. Therefore, an international guideline published in 2019 and many other studies recommend that therapeutic drug monitoring (TDM) should be performed for CMS to evaluate the drug exposure of its active ingredient, colistin, in patients [7,9].

To conduct TDM, it is essential to establish a sensitive, robust, and rapid method for the quantification of drug concentrations. Currently, the main methods for clinical detection of colistin and CMS include high-performance liquid chromatography (HPLC), HPLC Fluorescence, and liquid chromatography-tandem mass spectrometry (LC-MS/MS). Among them, LC-MS/MS is the most widely used method with superior sensitivity and specificity [[10], [11], [12]]. Current LC-MS/MS methods for the determination of CMS and colistin concentrations mainly use solid phase extraction (SPE) for sample preparation. After elution from a SPE column, some of the methods concentrate the eluate and then redissolve it prior to analysis [13]. One method injects the sample directly into the LC-MS/MS system after SPE, with a total run time of 15 min [14]. Additionally, some LC-MS/MS methods use protein precipitation for sample pretreatment and then detect the sample directly [15]. However, for various reasons, none of these methods can be considered highly satisfactory for clinical TDM applications. The reasons are as follows: 1) the sample preparation steps are cumbersome; or 2) the sample run time is relatively long; or 3) the sample processing is not sufficiently clean, leading to the risk of CMS degrading into colistin [12,16].

Drug stability is important for safe clinical administration and accurate monitoring of drug concentrations. Previous studies have shown that CMS is unstable in aqueous solutions and tends to convert to colistin in solution [17,18]. The degradation of CMS in infusion solutions may cause a toxicity risk to hospitalized patients. Additionally, CMS degradation during blood sample collection, transport, and pre-processing may affect the accurate determination of drug concentrations in patient samples [19,20]. Therefore, it is crucial to investigate the stability of CMS during infusion, sample collection, and processing procedures.

This study aims to establish a rapid, simple, and robust method for the quantification of colistin and CMS in human plasma. The developed method will be applied to TDM for colistin and CMS. Additionally, this study evaluated the stability of CMS in different clinical infusion solutions and blood collection procedures to ensure safe and effective infusion and accurate monitoring of blood concentrations.

## 2 Experimental

### 2.1 Chemicals and reagents

**Fig. 1:**
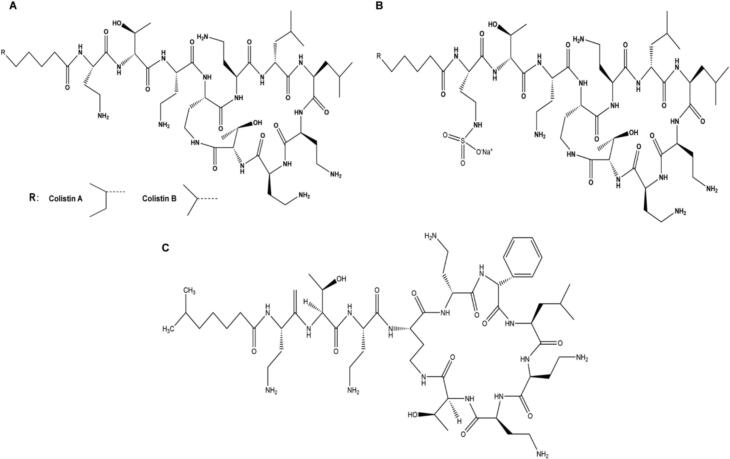
Molecular structure of colistin A and colistin B (A), CMS(B) and IS (C).

CMS (purity: 99.0 %) was acquired from Hubei Guangao Biotechnology Co., LTD (Hubei, China). Colistin A (purity: 99.9 %), colistin B (purity: 99.9 %), and polymyxin B2 (purity: 99.9 %) were purchased from TOKU-E (Bellingham, US). The molecular structure is shown in Fig. 1. Methanol and acetonitrile (HPLC grade) are purchased from Merck (Darmstadt, Germany). Formic acid (FA, HPLC grade) was purchased from Fuyu Fine (Tianjin, China). Ultrapure water was obtained from the PURELAB flex system (High Wycombe, UK). The solid phase extraction (SPE) column was Balanced Reverse Polymer (BRP) which is from Welch (Shanghai, China). Ammonia (25 %) was purchased from Guanghua (Guangdong, China). Blank plasma was provided by healthy volunteers (Ethical approval number: TJ-IRB20220737).

### 2.2 Chromatographic and mass spectrometry conditions

The LC-MS/MS system consists of AB SCIEX QTRAP 5500 (Boston, Massachusetts, USA) mass spectrometry and Shimadzu (Kyoto, Japan) liquid chromatography. The liquid chromatography is equipped with a CTO-20AC column oven (Kyoto, Japan) and a SIL-20ACHT auto-sampler (Kyoto, Japan).

Samples were separated on a Welch Ultimate LP-C18 column (4.6 × 100 mm, 5 μm) at a temperature of 40 °C. The mobile phase consisted of 1 % FA water (A) and 1 % Formic acid (FA) acetonitrile (B). The gradient elution program with a flow rate of 0.80 mL/min is as follows: 0 min, 8 % B; 0.5 min, 50 % B; 2 min,70 % B; 2.10 min, 95 % B; 3.50 min, 95 % B; 3.60 min, 8 % B; 5.00, stop. The injection volume was 10 µL.

**Fig. 2:**
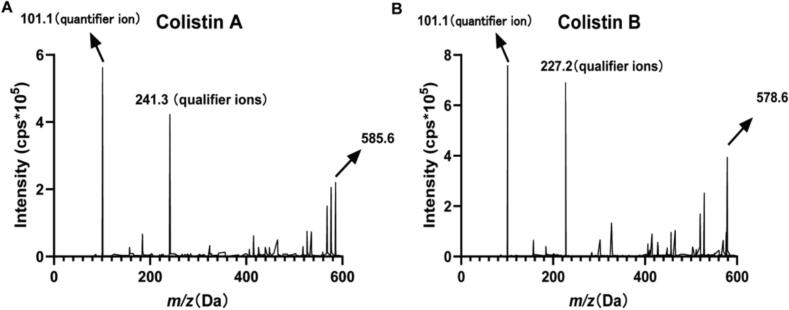
Mass spectra of colistin A (A) and colistin B (B).

The electrospray ionization model was employed for ionization, with positive ion mode used for ion scanning. The divalent ion [M + 2H]^2+^ was selected as the precursor ion for both the analytes and internal standard (IS). The SRM transitions monitored were *m*/*z* 585.6 → *m*/*z* 241.3 (qualifier ions) and *m*/*z* 585.6 → *m*/*z* 101.1 (quantifier ion) for colistin A, *m*/*z* 578.6 → *m*/*z* 227.2 (qualifier ions) and *m*/*z* 578.6 → *m*/*z* 101.1 (quantifier ion) for colistin B, and *m*/*z* 595.6 → *m*/*z* 101.1 for IS. The mass spectra of the analytes are provided (Fig. 2). After determining the corresponding production, optimization of the respective mass spectrometric parameters was carried out to maximize the mass spectrometric response. The optimal MS/MS setup parameters were a 40 psi collision gas flow (CUR); a 550°C desolvation temperature (TEM); a 60 psi gas flow 1; and a 60 psi gas flow 2. Other mass spectrometric parameters are given in Table 1.

**Table 1:** Mass spectrum parameters and linear coefficients of the compounds.

Compound	Precursor ion (*m*/*z*)	Product ion (*m*/*z*)	dwell time (ms)	DP (eV)	CE (eV)	Peak points	linear equation	R2	Concentration range (μg/mL)	LLOQ (μg/mL)
Colistin A	585.6	101.1	50	120	33.5	15	y = 0.000808x + 0.00701	0.9961	0.10–10	0.10
Colistin B	578.6	101.1	50	115	33.5	15	y = 0.000948x + 0.01864	0.9951	0.10–10	0.10
IS	595.6	101.1	40	150	32	15	NA			

### 2.3 Sample preparation

Colistin A and colistin B stock solutions were prepared at a concentration of 2,000 μg/mL, and the IS stock solution was a 2,000 μg/mL solution of polymyxin B2. All solutions were prepared using a methanol–water mixture (10:90, *v/v*) containing 0.1 % FA and stored at −80°C. The mixed working solution of colistin A and colistin B was prepared by diluting the respective stock solutions with the methanol–water mixture mentioned above. The calibration standards were prepared with working solutions at concentrations of 2, 5, 10, 20, 50, 100, 160, and 200 μg/mL. The concentrations of the QC working solutions, the lower limit of quantitation(LLOQ), low QC (LQC), medium QC (MQC), high QC (HQC), and dilution-reliability QC (DQC) were 2, 6, 40, 150, and 600 μg/mL, respectively. The IS working solution (4 μg/mL) was prepared by diluting the IS stock solution in methanol–water (50:50, *v/v*). The stock and working solutions were stored at −20°C.

The calibration standard samples and QC samples were both obtained by diluting the corresponding working solution with blank plasma at a ratio of 1:19. The concentrations of the calibration standard samples were 0.10, 0.25, 0.50, 1.0, 2.5, 5.0, 8.0 and 10 μg/mL while the concentrations of the QC samples were 0.10 (LLOQ), 0.30 (LQC), 2.0 (MQC), 7.5 (HQC), and 30 (DQC) μg/mL.

### 2.4 Determination of free colistin concentration

The SPE column was activated by using 1 mL of methanol followed by 2 mL of ultrapure water. Subsequently, 200 μL of either calibration standard samples, QC samples, or patient plasma samples were mixed with 100 μL of IS working solution and 700 μL of 1.43 % ammonia, This step is to ensure that the solution is in a non-acidic environment. This mixture was thoroughly vortexed to ensure homogeneity. The mixed samples were then loaded onto the Welch BRP SPE columns. After sequentially washing the column with a total of 4 mL of 1 % ammonia, the analytes were eluted twice with 200 μL of acetonitrile (30:70, *v/v*) containing 4 % formic acid. Ultimately, 400 μL total eluate was collected and analyzed by LC-MS/MS.

### 2.5 Determination of total colistin and CMS concentration

The concentration of CMS was determined by calculating the difference between the concentration of total colistin after complete *in vitro* conversion and the concentration of free colistin *in vivo*. *In vitro* conversion of CMS was performed by sulfuric acid hydrolysis. Samples were processed as follows: 25 μL of 1 mol/L H_2_SO_4_ was added to a tube containing 200 μL of patient plasma sample and 100 μL of IS working solution, acidified at room temperature on a shaker for 1 h, and then 50 μL of 1 mol/L NaOH was added to neutralize the acid hydrolysis to maintain the pH of the final solution in the range of 7.0–7.5. After vortex mixing, 625 μL of 1.43 % ammonia solution was added and mixed thoroughly. After this conversion process, the concentration of total colistin was determined as free colistin described above.

### 2.6 Method validation

According to the guidelines for quantitative analysis of biological samples from the U.S. Food and Drug Administration and the 2020 Chinese Pharmacopoeia, the methodology of this study is being validated [21,22]. The validation includes specificity, precision, accuracy, linearity, carryover, extraction recovery, matrix effect, dilution integrity, and stability.

#### 2.6.1 Specificity

Six different batches of blank human plasma from various sources were selected to assess the specificity of the analytical method. A comparison between the blank samples and the LLOQ samples was conducted to observe whether interference peaks were present at the retention time of the analytes and IS. The peak area of the interference peak should be less than 10 % of the peak area of the analytes at the LLOQ and the peak area of the interference peak should be less than 5 % of the IS peak area.

#### 2.6.2 Linearity

The calibration curves for colistin A and colistin B were evaluated linearly within the concentration range of 0.1 μg/mL to 10 μg/mL. Linear regression analysis was employed to fit the concentration of the analytes against the peak area ratio of the analyte to the IS. A calibration curve was established using a 1/x^^2^ weighting scheme. The accuracy of the back-calculated calibration concentrations should be within ±15 % or ±20 % (LLOQ) of the quantification limit, with at least 75 % of the calibration points meeting this accuracy requirement. The linear coefficient of the standard curve should be greater than 0.99.

#### 2.6.3 Precision and accuracy

Precision and accuracy were assessed by analyzing four levels of QC samples (LLOQ, LQC, MQC, and HQC) in sets of five replicates on three different days. Their concentrations were calibrated by calibration standards samples with known concentrations. The intra- and inter-day accuracy of LQC, MQC, and HQC should be within the range of 85 % to 115 %, The LLOQ sample should be within 80 % to 120 %. The intra- and inter-day precision, as indicated by the relative standard deviation (RSD) should be less than 15 %.

#### 2.6.4 Dilution integrity and carryover

Dilution integrity was assessed by dilution of DQC samples whose concentrations were above the upper limit of quantification (ULOQ) with blank plasma. Blank plasma was used to dilute the DQC sample 10-fold to obtain five repeated dilutions. After sample pre-treatment, the accuracy of the diluted samples should be within ±15 % and RSD should be ≤15 %. Carryover was conducted by injecting a blank sample after injecting the ULOQ sample. The peak area of the carryover in the blank sample should be less than 20 % of the peak area of the analyte in the LLOQ sample and should be less than 5 % of the peak area of the IS in other samples containing the IS.

#### 2.6.5 Extraction recovery and matrix effects

To assess matrix effects, a comparison was made between the peak areas of the analyte prepared in 4 % FA acetonitrile: water (30:70, *v/v*) and the peak areas of the analyte in the eluate from blank plasma after SPE. Extraction recovery was evaluated by comparing the peak areas of the analyte dissolved in the eluate from blank plasma after SPE and the peak areas of the analyte in the eluate from QC samples after SPE. Matrix effects and extraction recovery were both evaluated for LQC, MQC, and HQC samples at three different concentration levels, with five parallel samples prepared for each. The RSD for both matrix effects and extraction recovery across all concentration levels should be less than 15 %.

#### 2.6.6 Stability of colistin

This study investigated the stability of colistin A and colistin B during the assay process, including the stability of plasma samples at room temperature for 24 h, the stability through three cycles of repeated freeze–thaw from −80 °C to room temperature, the stability during storage at −80 °C for three months, and the autosampler stability of processed samples at 4°C for 24 h. Three parallel samples were prepared for each condition. If the accuracy of the samples under storage conditions is within 85 % to 115 %, the stability is considered acceptable.

### 2.7 Stability of CMS during clinical infusion and sample testing

CMS undergoes several steps from patient administration to final drug concentration monitoring, including infusion, blood collection, transport, sample preparation, analysis, and storage. During these processes, CMS degradation can compromise patient safety and concentration determination. We therefore investigated the stability of CMS in infusion solutions commonly used in clinical practice, including 0.9 % sodium chloride, 5 % dextrose injection, 10 % dextrose injection, 5 % glucose-sodium chloride, and lactated Ringer's solution at room temperature and 4°C. We also investigated the stability of CMS in whole blood and plasma samples at room temperature and on ice, as well as the long-term stability of CMS in plasma samples stored at −20°C.

### 2.8 Patient sample collection and TDM application

The study included three patients infected with carbapenem-resistant Gram-negative bacteria. The patients provided informed consent, and the research project was carried out following the regulations of the Ethics Committee (Ethical approval number: TJ-IRB20220737) of Tongji Medical College, Huazhong University of Science and Technology. The physician prescribed a weight-based dosing regimen with a 300 mg loading dose of colistin base activity (CBA) and a maintenance dose of 150 mg CBA. The sample collection for this session took place during the administration of the fourth dose. Blood samples were collected from the vein at pre-dose (0 h), at the end of the infusion (1 h), and 1, 3, 5, and 7 h after the end of the infusion. Blood samples were collected by EDTA tubes and placed on ice and transported to the lab within 2 h. The samples were centrifuged at a speed of 1000 g for 10 min at 4°C and the supernatant was stored at −20 °C for subsequent sample preparation.

Pharmacokinetic parameters, including area under the concentration–time curve from 0 to the last measurable time point (AUC_0-t_) and from 0 to infinity (AUC_0-∞_), peak concentration (C_max_), time to reach peak concentration (T_max_), elimination half-life (t_1/2_), apparent volume of distribution (V), apparent total clearance (CL), and mean residence time (MRT_0-∞_) were calculated using DAS 2.0 (Shanghai University of TCM, China).

## 3 Results and discussion

### 3.1 Method development

**Fig. 3:**
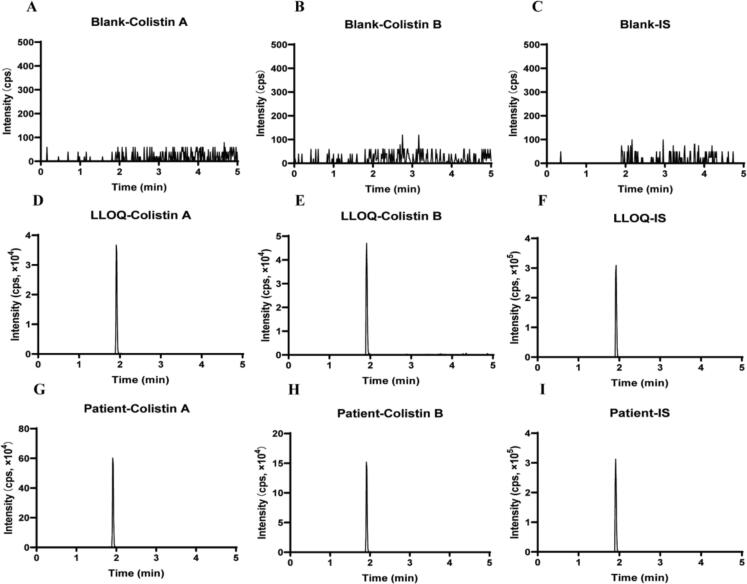
Representative chromatograms of blank plasma samples (A, B, C), plasma samples spiked with colistin A and colistin B at the LLOQ and the internal standard (D, E, F) and a representative patient sample with colistin A (1.64 μg/mL), colistin B (0.27 μg/mL) and the internal standard (G, H, I).

Methodological exploration was carried out using different chromatographic columns, including the Waters SunFire C18 (3.5 μm, 4.6 × 150 mm), the Agilent XDB-C18 column (5 μm, 4.6 × 250 mm), and the Welch LP-C18 column (5 μm, 4.6 × 100 mm) and different aqueous phases, containing several proportions of FA (0 %, 0.1 %, 0.5 %, 1 %) and different organic phases (i.e., Methanol, acetonitrile, or combinations of methanol and acetonitrile at different ratios). The peak characteristics of the analytes and IS, as well as the mass spectrometric response under each condition were carefully analyzed and examined. The results showed that better peak shapes and mass spectrometric responses were observed with the Welch LP-C18 chromatographic column with ultrapure water containing 1 % FA and acetonitrile containing 1 % FA as the mobile phase [14,15,23]. The optimized method developed a gradient elution program with a duration of 5 min, which is shorter than that of several other established methods [15,24]. Colistin A, colistin B, and the IS all eluted at 1.93 min with symmetrical and narrow peak shapes (Fig. 3). Five replicate injections yielded nearly identical peak areas with an RSD of 0.71 %, indicating that the method has good reproducibility and meets the analytical requirements. The theoretical plate count reached 7,048 (>2,000). Throughout the 5-minute runtime, no co-eluting peaks or baseline interference was observed. The target peak exhibited ideal symmetry with a tailing factor of 1.02 (acceptable range: 0.95–1.05), showing neither fronting nor tailing anomalies (Fig. 3).

The inherent instability of CMS imposes stringent requirements on sample pre-treatment methods. In preliminary studies, we found that conventional methods such as protein precipitation (PP) and liquid–liquid extraction (LLE) were unable to prevent the degradation of CMS (data not shown). This degradation introduces a significant bias in the measurement of colistin concentration. In contrast, SPE can effectively elute CMS from plasma samples, thus eliminating the interference of CMS degradation [23,25]. Therefore, the SPE column was selected as the sample pre-processing method. Next, actual samples were used to verify whether SPE could completely clear CMS. The processed patient samples were again subjected to acid hydrolysis followed by chromatographic analysis. As shown in Supplementary Fig. 1, no significant concentration differences were observed between the hydrolyzed and non-hydrolyzed groups, indicating that SPE was effective in removing CMS from the samples [23]. Oasis HLB, Oasis WCX, and Welch BRP columns were tested in this study. After evaluating the recovery, reproducibility, and price, the Welch BRP column was used for sample pre-processing, with excellent extraction recovery (91.93 %-100.9 %), remarkable repeatability (RSD, 6.14 %), and low cost. Compared with the method established by Leporati M et al and Qin Fu et al [26,27]. the sample pretreatment used in this study does not require the concentration of eluent, which greatly shortens the pre-processing time and is beneficial for large-scale clinical testing.

### 3.2 Method validation

The analyte and IS peaks were symmetrical and no peaks that interfered with either the analyte or IS were found in the blank plasma, indicating that this method has high specificity. The chromatograms of blank plasma samples, LLOQ samples, and patient samples are shown in Fig. 3.

Calibration curves were plotted against concentration using the peak areas ratio of colistin A and colistin B to the IS. The calibration curves were fitted using weighted (1/x^^2^) least squares linear regression. The linear regression equations for colistin A and colistin B are presented in Table 1. The linear range for both colistin A and colistin B was 0.1–10 μg/mL, which covers the actual patient concentrations reported (0.24–9.92 μg/mL) [28,29]. The linear correlation coefficients (R^2^) of all curves were greater than 0.995.

The intra-day and inter-day accuracy for LLOQ, LQC, MQC, and HQC samples of colistin A and colistin B ranged between 90.97 % and 114.65 %. The intra-day precision was less than 6.17 % and the inter-day precision was less than 10.69 % (Table 2). Furthermore, the dilution reliability of high-concentration DQC samples following a 10-fold dilution with blank plasma was studied. The accuracy and precision of the diluted samples were within the range of 94.41 % to 113.95 % and below 8.42 %, respectively. There was no carryover of analytes or IS in newly injected blank plasma samples after injecting ULOQ samples.

**Table 2:** Intra- and inter-assay accuracy and precision results of colistin A and colistin B (n = 6).

Analyte	Quality control sample	Sample concentration (μg/mL)	Intra-dayAccuracy/%	Inter-dayAccuracy/%	Intra-dayprecision RSD/%	Inter-dayprecision RSD/%
Colistin A	LLOQ	0.10	92.96 ± 2.87	97.92 ± 4.26	3.01	7.79
	LQC	0.30	114.65 ± 5.82	102.33 ± 7.44	5.07	2.52
	MQC	2.0	92.48 ± 3.25	93.57 ± 7.76	3.51	8.29
	HQC	7.5	107.83 ± 6.65	94.00 ± 9.67	6.17	10.29

Colistin B	LLOQ	0.10	93.56 ± 4.78	99.21 ± 8.41	5.10	8.48
	LQC	0.30	107.36 ± 1.45	105.12 ± 4.82	1.35	5.57
	MQC	2.0	90.97 ± 4.67	93.29 ± 8.81	5.14	10.07
	HQC	7.5	102.86 ± 5.18	94.02 ± 7.72	5.04	8.21

Matrix effects on the analytes and IS were assessed by evaluating blank plasma from six batches of different healthy subjects (Ethical approval number: TJ-IRB20220737) (Table 3). The relative matrix effects for colistin A at low, medium, and high QC levels ranged from 0.96 to 1.03, while for colistin B, the relative matrix effects at the same three levels ranged from 0.98 to 1.00. The RSD for colistin A and colistin B in each of these six batches of different blank plasma was less than 6 %. The relative extraction recovery of colistin A and colistin B for three QC levels was 91.93 % to 100.93 % and 92.91 % to 94.30 %, respectively. The recovery RSD for the three different QC levels was all <15 %.

**Table 3:** Matrix effect and extraction recovery of colistin A, colistin B, and IS (n = 6).

Analyte	Relative matrix effect /%			RSD /%	Relative recovery /%			RSD /%
	0.10 (μg/mL)	2.0 (μg/mL)	7.5 (μg/mL)		0.10 (μg/mL)	2.0 (μg/mL)	7.5 (μg/mL)	
Colistin A	95.60 ± 5.90	99.79 ± 3.57	103.09 ± 4.28	5.9	100.93 ± 4.52	91.93 ± 3.44	96.68 ± 6.14	6.14
Colistin B	100.08 ± 3.91	98.18 ± 4.41	99.60 ± 2.96	3.91	94.30 ± 5.48	92.91 ± 5.06	93.22 ± 5.02	5.06

The stability results for colistin are presented in Table 4. The results show that plasma samples of colistin A and colistin B remain stable when left at room temperature for 24 h, subjected to three cycles of repeated freeze–thaw from −80 °C to room temperature, and stored at −80 °C for three months. Processed colistin samples can be stable for 24 h when stored at 4°C, suggesting that this method is suitable for handling a large number of samples over an extended period.

**Table 4:** Stability of colistin A and colistin B (n = 3).

Analyte	QC sample	Theoretical concentration (μg/mL)	−80 °C freeze–thaw 3 times /%	Frozen at −80 °C for 3 months /%	Room temperature 24 h /%	Processed sample In the sampler at 4 °C for 24 h /%
Colistin A	LQC	0.30	106.19 ± 2.48	98.31 ± 5.04	104.77 ± 4.59	102.44 ± 1.43
	HQC	7.5	93.04 ± 1.97	101.24 ± 1.46	91.48 ± 3.06	100.74 ± 4.06

Colistin B	LQC	0.30	110.17 ± 4.65	109.29 ± 3.71	110.01 ± 3.95	108.54 ± 3.07
	HQC	7.5	99.13 ± 6.46	98.76 ± 4.29	99.39 ± 1.70	106.46 ± 6.48

### 3.3 Stability of CMS during clinical infusion and sample testing

**Fig. 4:**
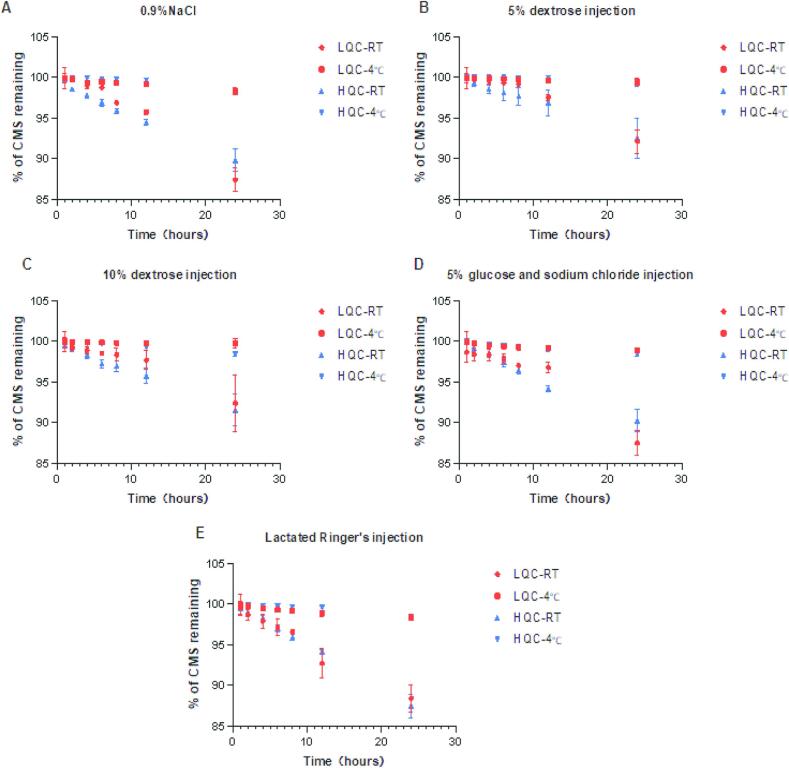
The stability of CMS in various clinical infusion solutions, including **(A)** 0.9% sodium chloride injection, **(B)** 5% dextrose injection, **(C)** 10% dextrose injection, **(D)** 5% glucose and sodium chloride injection, and **(E)** Lactated Ringer's injection, when stored at room temperature and 4 °C for 0–24 h. (Notes: RT, room temperature).

CMS degradation before infusion may cause toxicity in hospitalized patients [19,20], and CMS degradation in collected blood and plasma samples may affect the accurate detection of colistin concentration. Therefore, understanding the stability of CMS in infusion solutions, whole blood, and plasma samples is crucial. The stability of CMS in commonly-used infusion solutions including 0.9 % sodium chloride, 5 % dextrose injection, 10 % dextrose injection, 5 % glucose-sodium chloride, and lactated Ringer's solution was investigated at concentrations likely to be used in clinical practice (i.e., 0.5 μg/mL and 1.5 μg/mL). The results showed that CMS remained stable (degradation rate <5%) when left at room temperature for 8 h or stored at 4°C for 24 h at any infusion solutions. However, significant degradation of CMS was observed in all five infusion solutions after 24 h of storage at room temperature, with degradation rates ranging from 8 % to 13 % (Fig. 4). Therefore, intravenous infusion of CMS should ideally be prepared and used immediately, or pre-prepared infusion solutions should be stored at 4 °C.

**Fig. 5:**
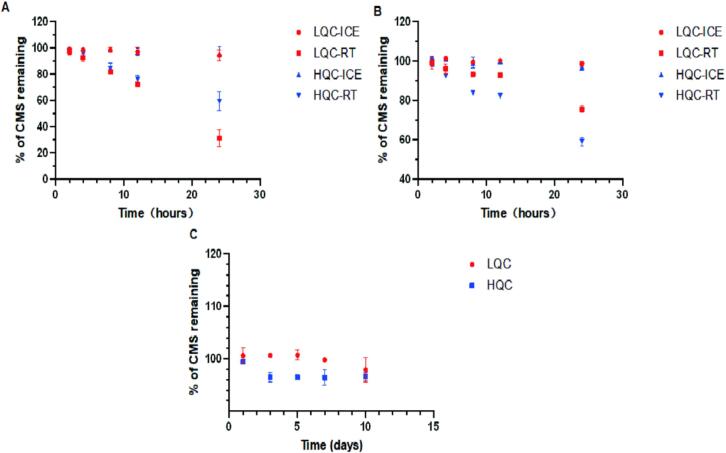
The stability of CMS in whole blood and plasma samples. **(A)** The stability of CMS in whole blood under room temperature and ice conditions for 0–24 h. **(B)** The stability of CMS in blank plasma under room temperature and ice conditions for 0–24 h. **(C)** The stability of CMS in blank plasma when stored at −20 °C for 0 to 10 days.

Next, we investigated the stability of CMS in whole blood samples at two concentration levels (5 μg/mL and 50 μg/mL) when stored at room temperature and on ice (Fig. 5). After 8 h of storage at room temperature, the degradation of CMS exceeded 15 % [18,30]. However, when CMS blood samples were stored on ice for 24 h, the maximum degradation rate of CMS was only 3.18 %, significantly lower than the degradation rate observed under room temperature conditions (as high as 40.94 % over the same period). In addition, we also studied the stability of CMS in plasma samples, and the results showed similar stability results to that of CMS in whole blood. Thus, transport and pre-processing of CMS samples on ice is a preferred option to prevent CMS degradation. Lastly, the long-term stability of CMS in plasma samples was investigated and the degradation rate of CMS was less than 5 % when stored at −20°C for 10 days (Fig. 5).

### 3.4 Clinical TDM application

**Fig. 6:**
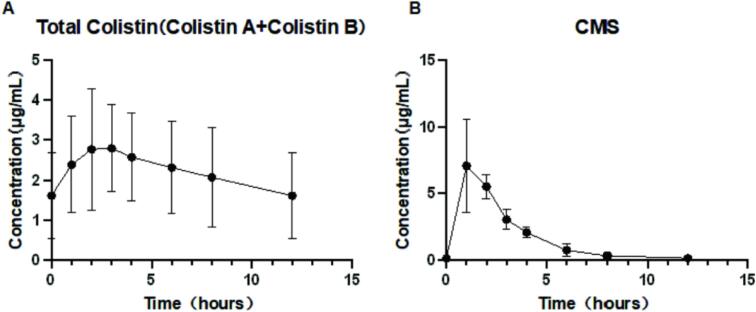
Plasma concentration–time curve of CMS and colistin in patient samples.

The concentration–time curves and PK parameters of colistin and CMS are shown in Fig. 6 and Table 5, respectively. A total of three patients treated with CMS for carbapenem-resistant Gram-negative bacterial infections were included in this study (Ethical approval number: TJ-IRB20220737). The measured trough concentrations of colistin (C_min_) ranged from 0.97 μg/mL to 2.84 μ g/mL, and the average C_min_ was 1.61 ± 1.06 μ g/mL. CMS achieved peak concentration (C_max_) at the end of the infusion, whereas colistin reached C_max_ at 1-hour post-infusion, suggesting that the conversion of CMS to colistin *in vivo* is relatively slow. This result is also consistent with the conclusions in the study of healthy subjects (T_max-CMS_ = 1 h, T_max-colistin_ = 2 h) [31].

**Table 5:** The PK parameters of the patient.

PK parameters		**Patient**							
		**Value**^a^		**1**		**2**		**3**	
		CMS	Colistin	CMS	Colistin	CMS	Colistin	CMS	Colistin
AUC_0-12h_	mg·h/L	22.08 ± 4.54	26.75 ± 14.10	21.20	18.73	26.99	43.03	18.03	18.49
AUC_0-24h_	mg·h/L	43.49 ± 8.55	53.43 ± 27.98	41.04	37.48	53.00	85.74	36.44	37.08
AUC_0-∞_	mg·h/L	22.46 ± 4.71	53.93 ± 44.49	21.53	31.44	27.57	105.17	18.29	25.18
C_min_	mg/L	0.00	1.61 ± 1.06	0.00	0.97	0.00	2.84	0.00	1.04
C_max_	mg/L	8.21 ± 1.52	3.01 ± 1.32	8.79	2.08	9.35	4.52	6.49	2.45
C_ss_	mg/L	1.81 ± 0.36	2.23 ± 1.17	1.71	1.56	2.21	3.57	1.52	1.55
T_max_	hour	1.33 ± 0.58	2.33 ± 0.58	1.00	2.00	1.00	2.00	2.00	3.00
Vz	L	14.11 ± 2.39	47.53 ± 15.89	12.54	62.61	16.86	30.93	12.92	49.04
t_1/2_	hour	1.50 ± 0.57	9.94 ± 4.72	1.25	9.09	2.15	15.03	1.09	5.71
CL	L/h	6.87 ± 1.38	4.05 ± 2.35	6.97	4.77	5.44	1.43	8.2	5.96
MRT_0-∞_	hour	2.99 ± 0.78	15.41 ± 5.87	2.21	13.34	3.77	22.04	2.98	10.86

a Values are mean ± SD.

In this study, the calculated half-life (t_1/2_) of colistin and CMS was 9.94 ± 4.72 h and 1.50 ± 0.57 h, respectively. In the study by Couet W et al. in healthy volunteers, the half-life of colistin and CMS was 3.0 ± 0.6 h and 2.0 ± 0.12 h, respectively. In studies of critically ill patients, the half-life of colistin ranges from 9.8 to 14.4 h, and the half-life of CMS ranges from 2.1 to 2.3 h [31,32]. Compared to healthy subjects, the half-life of colistin in the patients in this study is closer to that observed in critically ill patients, while the half-life of CMS shows no significant difference.

The colistin AUC_0-24h_ values for the three patients were 37.43, 86.05, and 36.98 mg∙h/L, respectively, with corresponding C_ss,avg_ values of 1.56, 3.59, and 1.54 mg/L. Nation et al. observed a C_ss,avg_ range of 0.24–9.92 μg/mL during the interdose interval (days 3–5 of therapy) in critically ill patients [28]. Similarly, Leuppi-Taegtmeyer et al. reported concentrations ranging from 2.13 to 7.24 μg/mL in patients undergoing renal replacement therapy (RRT) who were dosed as if they had normal renal function [33]. In pediatric critically ill patients, Charalampos et al. documented a C_ss,avg_ range of 1.11–8.47 μg/mL [34]. Based on other studies and our results, there was significant interpatient variability in colistin exposure.

Current clinical consensus and several other studies recommend a target steady-state blood drug concentration (C_ss,avg_) of 2 mg/L, corresponding to an AUC_0-24h_ = 50 mg∙h/L, as a therapeutic goal for colistin [9,28]. If the C_ss,avg_ is greater than 2 μg/mL, the risk and severity of acute renal failure are significantly increased [9,35]. In this study, two patients failed to achieve therapeutic exposure (AUC_0-24h_ = 37.43 and 36.98 mg·h/L, respectively), while another patient was as high as 86.05 mg/L with a high risk of drug toxicity.

In several published studies, blood samples were typically taken before administration, immediately after completion of the infusion, and at several subsequent time points (e.g., 0.5, 1, 2, 4, 8 h), usually 4–8 blood sampling points, to fully characterise the pharmacokinetics of colistin and CMS [36,37]. Our blood sampling protocol was also largely consistent with the above protocol [38]. Notably, research highlights the 2-hour post-dose timepoint as particularly critical for calculating AUC_0-24h_ due to its strong correlation with total drug exposure [39,40]. Therefore, it is necessary to establish an optimized sampling strategy for precise AUC determination in clinical practice.

Given the narrow therapeutic window and significant inter-individual PK variability, it is critical to establish a robust analytical method and blood sampling strategy for TDM of colistin in patients to optimize both safety and efficacy [31].

## 4 Conclusion

The method presented in this study is robust, simpler, and faster compared to the previously published methods using SPE to prepare samples and LC-MS/MS to quantify colistin and CMS in plasma. The stability of CMS during infusion and detection was thoroughly investigated. Furthermore, this method will be beneficial to be applied in the future to perform TDM of CMS and colistin in critically ill patients.

## Ethics statement

This study included three patients infected with carbapenem-resistant Gram-negative bacteria and healthy volunteers who provided blank plasma. All provided informed consent and the research project was carried out following the regulations of the Ethics Committee (Ethical approval number: TJ-IRB20220737) of Tongji Medical College, Huazhong University of Science and Technology.

## Funding sources

Xuepeng Gong was supported by the Natural Science Foundation of Hubei Province (NO.2022CFB142) and Dong Xiang was supported by the National Natural Science Foundation of China (NO. 82104507).

## CRediT authorship contribution statement

**Tinghui Zhao:** Writing – review & editing, Writing – original draft, Validation, Methodology. **Lu Liu:** Writing – review & editing, Supervision. **Guangjie Yang:** Methodology, Investigation. **Hengyi Yu:** Writing – review & editing, Formal analysis. **Lihui Qiu:** Methodology, Investigation, Conceptualization. **Xiping Li:** Writing – review & editing, Software, Methodology. **Dong Xiang:** Writing – review & editing, Supervision, Software, Methodology, Investigation, Conceptualization. **Xuepeng Gong:** Writing – review & editing, Supervision.

## Declaration of competing interest

The authors declare that they have no known competing financial interests or personal relationships that could have appeared to influence the work reported in this paper.

## References

[1] Bialvaei A.Z., Samadi Kafil H. (2015). Colistin, mechanisms and prevalence of resistance. Curr. Med. Res. Opin..

[2] Karaiskos I., Giamarellou H. (2014). Multidrug-resistant and extensively drug-resistant gram-negative pathogens: current and emerging therapeutic approaches. Expert Opin. Pharmacother..

[3] Falagas M.E., Kasiakou S.K. (2006). Toxicity of polymyxins: a systematic review of the evidence from old and recent studies. Crit. Care.

[4] Li J., Nation R.L. (2006). Old polymyxins are back: is resistance close?. Clin. Infect. Dis..

[5] Van den Bossche L., Van Schepdael A., Chopra S., Hoogmartens J., Adams E. (2011). Identification of impurities in polymyxin B and colistin bulk sample using liquid chromatography coupled to mass spectrometry. Talanta.

[6] Bergen P.J., Li J., Rayner C.R., Nation R.L. (2006). Colistin methanesulfonate is an inactive prodrug of colistin against Pseudomonas aeruginosa. Antimicrob. Agents Chemother..

[7] Landersdorfer C.B., Nation R.L. (2015). Colistin: how should it be dosed for the critically ill?. Semin. Respir. Crit. Care Med..

[8] Bergen P.J., Landersdorfer C.B., Lee H.J., Li J., Nation R.L. (2012). 'Old' antibiotics for emerging multidrug-resistant bacteria. Curr. Opin. Infect. Dis..

[9] B.T. Tsuji, J.M. Pogue, A.P. Zavascki, M. Paul, G.L. Daikos, A. Forrest, D.R. Giacobbe, C. Viscoli, H. Giamarellou, I. Karaiskos, D. Kaye, J.W. Mouton, V.H. Tam, V. Thamlikitkul, R.G. Wunderink, J. Li, R.L. Nation, K.S. Kaye, International Consensus Guidelines for the Optimal Use of the Polymyxins: Endorsed by the American College of Clinical Pharmacy (ACCP), European Society of Clinical Microbiology and Infectious Diseases (ESCMID), Infectious Diseases Society of America (IDSA), International Society for Anti-infective Pharmacology (ISAP), Society of Critical Care Medicine (SCCM), and Society of Infectious Diseases Pharmacists (SIDP), Pharmacotherapy 39 (2019) 10–39. doi: 10.1002/phar.2209. 10.1002/phar.2209 PMC7437259 30710469

[10] Ouchi S., Matsumoto K., Okubo M., Yokoyama Y., Kizu J. (2018). Development of HPLC with fluorescent detection using NBD-F for the quantification of colistin sulfate in rat plasma and its pharmacokinetic applications. Biomed. Chromatogr..

[11] Papavasileiou K., Tsiasioti A., Tzanavaras P.D., Zacharis C.K. (2022). HPLC determination of colistin in human urine using alkaline mobile phase combined with post-column derivatization: validation using accuracy profiles. Molecules.

[12] Jansson B., Karvanen M., Cars O., Plachouras D., Friberg L.E. (2009). Quantitative analysis of colistin A and colistin B in plasma and culture medium using a simple precipitation step followed by LC/MS/MS. J. Pharm. Biomed. Anal..

[13] Xu Y., Tian X., Ren C., Huang H., Zhang X., Gong X., Liu H., Yu Z., Zhang L. (2012). Analysis of colistin A and B in fishery products by ultra performance liquid chromatography with positive electrospray ionization tandem mass spectrometry. J. Chromatogr. B Analyt. Technol. Biomed. Life Sci..

[14] Dotsikas Y., Markopoulou C.K., Koundourellis J.E., Loukas Y.L. (2011). Validation of a novel LC-MS/MS method for the quantitation of colistin A and B in human plasma. J. Sep. Sci..

[15] Kim K.Y., Kim B.H., Kwack W.G., Kwon H.J., Cho S.H., Kim C.W. (2023). Simple and robust LC-MS/MS method for quantification of colistin methanesulfonate and colistin in human plasma for therapeutic drug monitoring. J. Pharm. Biomed. Anal..

[16] Qi B., Gijsen M., Van Brantegem P., De Vocht T., Deferm N., Abza G.B., Nauwelaerts N., Wauters J., Spriet I., Annaert P. (2020). Quantitative determination of colistin A/B and colistin methanesulfonate in biological samples using hydrophilic interaction chromatography tandem mass spectrometry. Drug Test. Anal..

[17] Siegenthaler-Zuber G. (1976). Which uric acid value is in need of treatment?. Schweiz. Med. Wochenschr..

[18] Wallace S.J., Li J., Rayner C.R., Coulthard K., Nation R.L. (2008). Stability of colistin methanesulfonate in pharmaceutical products and solutions for administration to patients. Antimicrob. Agents Chemother..

[19] Santamaria C., Mykietiuk A., Temporiti E., Stryjewski M.E., Herrera F., Bonvehi P. (2009). Nephrotoxicity associated with the use of intravenous colistin. Scand. J. Infect. Dis..

[20] McCoy K.S. (2007). Compounded colistimethate as possible cause of fatal acute respiratory distress syndrome. N. Engl J. Med..

[21] Food and Drug Administration, Guidance for Industry: Bioanalytical Method Validation. (2001).

[22] N. P. Commission, Chinese Pharmacopoeia, Beijing: China Medical Science Press Volume IV (2020) 480.

[23] Zhao M., Wu X.J., Fan Y.X., Guo B.N., Zhang J. (2016). Development and validation of a UHPLC-MS/MS assay for colistin methanesulphonate (CMS) and colistin in human plasma and urine using weak-cation exchange solid-phase extraction. J. Pharm. Biomed. Anal..

[24] Barco S., Castagnola E., Mesini A., Tripodi G., Cangemi G. (2019). Potential pitfalls in LC-MS/MS quantification of colistin for therapeutic drug monitoring of patients treated with colistimethate. J. Pharm. Biomed. Anal..

[25] Ma Z., Wang J., Gerber J.P., Milne R.W. (2008). Determination of colistin in human plasma, urine and other biological samples using LC-MS/MS. J. Chromatogr. B Analyt. Technol. Biomed. Life Sci..

[26] Leporati M., Bua R.O., Mariano F., Carignano P., Stella M., Biancone L., Vincenti M. (2014). Determination by LC-MS/MS of colistins A and B in plasma and ultrafiltrate from critically ill patients undergoing continuous venovenous hemodiafiltration. Ther. Drug Monit..

[27] Fu Q., Li X., Zheng K., Ke Y., Wang Y., Wang L., Yu F., Xia X. (2018). Determination of colistin in animal tissues, egg, milk, and feed by ultra-high performance liquid chromatography-tandem mass spectrometry. Food Chem..

[28] Nation R.L., Garonzik S.M., Thamlikitkul V., Giamarellos-Bourboulis E.J., Forrest A., Paterson D.L., Li J., Silveira F.P. (2017). Dosing guidance for intravenous colistin in critically-ill patients. Clin. Infect. Dis..

[29] Mohamed A.F., Karaiskos I., Plachouras D., Karvanen M., Pontikis K., Jansson B., Papadomichelakis E., Antoniadou A., Giamarellou H., Armaganidis A., Cars O., Friberg L.E. (2012). Application of a loading dose of colistin methanesulfonate in critically ill patients: population pharmacokinetics, protein binding, and prediction of bacterial kill. Antimicrob. Agents Chemother..

[30] Dudhani R.V., Nation R.L., Li J. (2010). Evaluating the stability of colistin and colistin methanesulphonate in human plasma under different conditions of storage. J. Antimicrob. Chemother..

[31] Couet W., Gregoire N., Gobin P., Saulnier P.J., Frasca D., Marchand S., Mimoz O. (2011). Pharmacokinetics of colistin and colistimethate sodium after a single 80-mg intravenous dose of CMS in young healthy volunteers. Clin. Pharmacol. Ther..

[32] Gregoire N., Mimoz O., Megarbane B., Comets E., Chatelier D., Lasocki S., Gauzit R., Balayn D., Gobin P., Marchand S., Couet W. (2014). New colistin population pharmacokinetic data in critically ill patients suggesting an alternative loading dose rationale. Antimicrob. Agents Chemother..

[33] Leuppi-Taegtmeyer A.B., Decosterd L., Osthoff M., Mueller N.J., Buclin T., Corti N. (2019). Multicenter population Pharmacokinetic study of colistimethate sodium and colistin dosed as in normal renal function in patients on continuous renal replacement therapy. Antimicrob. Agents Chemother..

[34] Antachopoulos C., Geladari A., Landersdorfer C.B., Volakli E., Ilia S., Gikas E., Gika H., Sdougka M., Nation R.L., Roilides E. (2021). Population Pharmacokinetics and outcomes of critically ill pediatric patients treated with intravenous colistin at higher than recommended doses. Antimicrob. Agents Chemother..

[35] Horcajada J.P., Sorli L., Luque S., Benito N., Segura C., Campillo N., Montero M., Esteve E., Mirelis B., Pomar V., Cuquet J., Marti C., Garro P., Grau S. (2016). Validation of a colistin plasma concentration breakpoint as a predictor of nephrotoxicity in patients treated with colistin methanesulfonate. Int. J. Antimicrob. Agents.

[36] Ooi M.H., Ngu S.J., Chor Y.K., Li J., Landersdorfer C.B., Nation R.L. (2019). Population pharmacokinetics of intravenous colistin in pediatric patients: implications for the selection of dosage regimens. Clin. Infect. Dis..

[37] Koomanachai P., Landersdorfer C.B., Chen G., Lee H.J., Jitmuang A., Wasuwattakul S., Sritippayawan S., Li J., Nation R.L., Thamlikitkul V. (2014). Pharmacokinetics of colistin methanesulfonate and formed colistin in end-stage renal disease patients receiving continuous ambulatory peritoneal dialysis. Antimicrob. Agents Chemother..

[38] Jacobs M., Gregoire N., Megarbane B., Gobin P., Balayn D., Marchand S., Mimoz O., Couet W. (2016). Population Pharmacokinetics of colistin methanesulfonate and colistin in critically ill patients with acute renal failure requiring intermittent hemodialysis. Antimicrob. Agents Chemother..

[39] Dudhani R.V., Turnidge J.D., Coulthard K., Milne R.W., Rayner C.R., Li J., Nation R.L. (2010). Elucidation of the pharmacokinetic/pharmacodynamic determinant of colistin activity against Pseudomonas aeruginosa in murine thigh and lung infection models. Antimicrob. Agents Chemother..

[40] Kim E.J., Oh J., Lee K., Yu K.S., Chung J.Y., Hwang J.H., Nam E.Y., Kim H.S., Kim M., Park J.S., Song K.H., Kim E.S., Song J., Kim H.B. (2019). Pharmacokinetic characteristics and limited sampling strategies for therapeutic drug monitoring of colistin in patients with multidrug-resistant gram-negative bacterial infections. Ther. Drug Monit..

