# Supplementary material for: Development of an LC-MS/MS method for quantification of colistin and colistin methanesulfonate in human plasma and its application to stability studies and therapeutic drug monitoring
Source: J Mass Spectrom Adv Clin Lab. 2025 Jun 1;37:39–48. doi: 10.1016/j.jmsacl.2025.05.001 (PMC12179625; doi:10.1016/j.jmsacl.2025.05.001)
Supplement: Supplementary Data 1 [file mmc1.docx]

| **Supplementary Table 1**. Calibration Curve Validation | | | | |
| --- | --- | --- | --- | --- |
| Nominal Concentration (μg/mL) | | Mean Measured Concentration (μg/mL) | Accuracy (%) | RSD (%) |
| Colistin A (n=3) | 0.10（LLOQ） | 0.10 | 103.62 | 9.72 |
|  | 0.25 | 0.25 | 98.29 | 2.18 |
|  | 0.50 | 0.50 | 100.13 | 7.49 |
|  | 1.0 | 1.02 | 102.46 | 8.58 |
|  | 2.5 | 2.58 | 103.20 | 4.29 |
|  | 5.0 | 4.86 | 97.24 | 3.34 |
|  | 8.0 | 7.75 | 96.92 | 0.42 |
|  | 10 | 10.14 | 101.40 | 10.57 |
|  |  |  |  |  |
| Colistin B (n=3) | 0.10（LLOQ） | 0.10 | 101.95 | 7.59 |
|  | 0.25 | 0.26 | 105.78 | 3.04 |
|  | 0.50 | 0.50 | 99.39 | 3.58 |
|  | 1.0 | 1.01 | 100.79 | 10.00 |
|  | 2.5 | 2.54 | 101.79 | 4.10 |
|  | 5.0 | 5.13 | 102.61 | 0.38 |
|  | 8.0 | 7.66 | 95.81 | 6.84 |
|  | 10 | 9.61 | 96.14 | 0.36 |

| **Supplement Table 2.** Standard solution and quality control solution preparation table | | | | | | | | |
| --- | --- | --- | --- | --- | --- | --- | --- | --- |
| Solution Code | Source Solution Code | Source Concentration (μg/mL) | Source Volume (μL) | Diluent | Diluent Volume (μL) | Final Volume (μL) | Final Concentration (μg/mL) | Notes |
| **C01** | NA | | | | | | 200 | Calibration Standard |
| **C02** | C01 | 200 | 200 | 0.2% FA in ultrapure water | 50 | 250 | 160 | Calibration Standard |
| **C03** | C01 | 200 | 100 | 0.2% FA in ultrapure water | 100 | 200 | 100 | Calibration Standard |
| **C04** | C01 | 200 | 100 | 0.2% FA in ultrapure water | 300 | 400 | 50 | Calibration Standard |
| **C05** | C01 | 200 | 100 | 0.2% FA in ultrapure water | 900 | 1000 | 20 | Calibration Standard |
| **C06** | C05 | 20 | 100 | 0.2% FA in ultrapure water | 100 | 200 | 10 | Calibration Standard |
| **C07** | C05 | 20 | 100 | 0.2% FA in ultrapure water | 300 | 400 | 5 | Calibration Standard |
| **C08** | C05 | 20 | 100 | 0.2% FA in ultrapure water | 900 | 1000 | 2 | Calibration Standard |
| **HQC** | NA | | | | | | 150 | High-Quality Control |
| **MQC** | HQC | 200 | 300 | 0.2% FA in ultrapure water | 825 | 1125 | 40 | Mid-Quality Control |
| **LQC** | HQC | 200 | 40 | 0.2% FA in ultrapure water | 960 | 1000 | 6 | Low-Quality Control |
| **DQC** | NA | | | | | | 300 | Dilution Quality Control |

NA:The solutions here were directly prepared by mixing colistin A stock solution and colistin B stock solution with a diluent (0.2% formic acid in ultrapure water) in varying proportions.


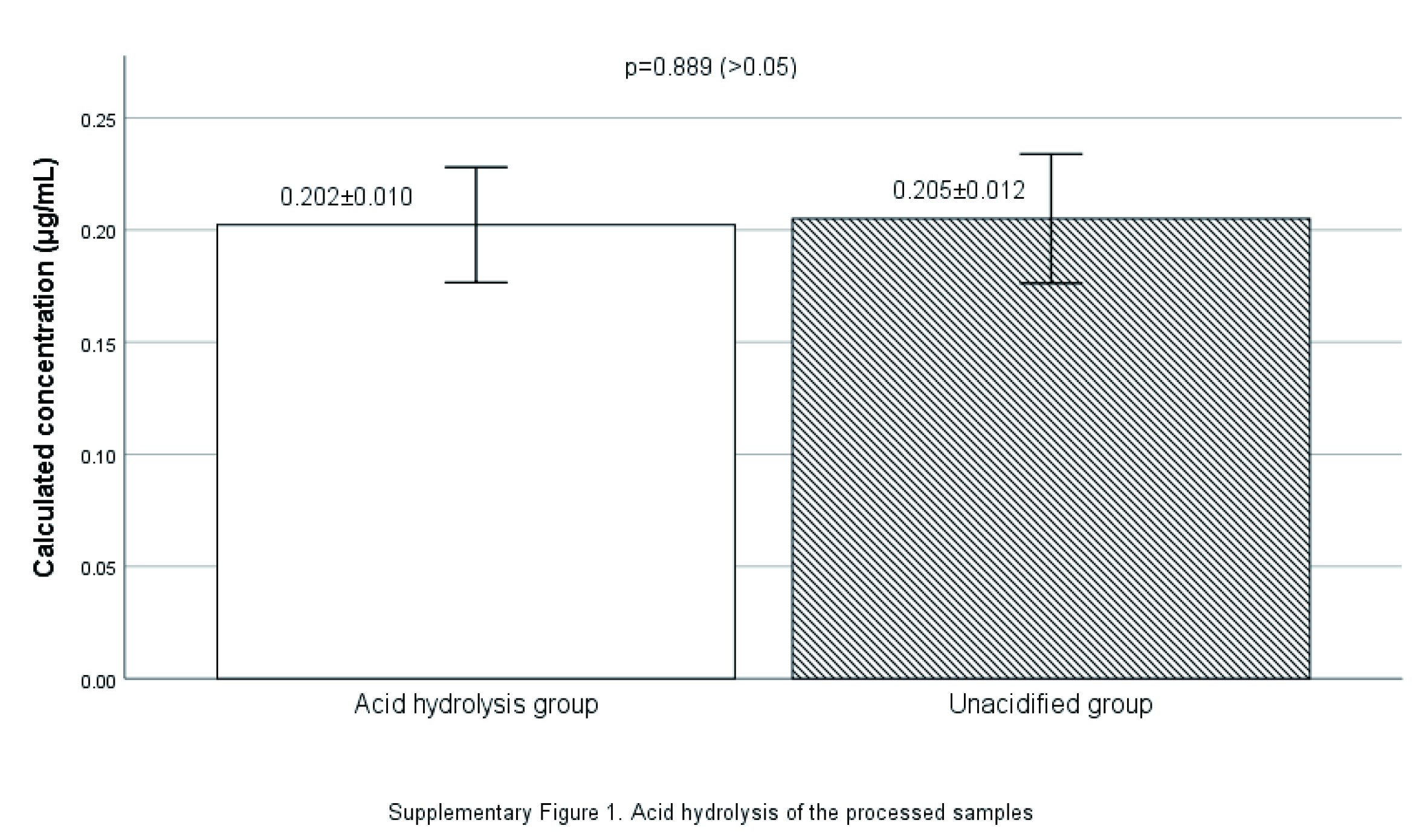


**Supplemental Figure 1** Acid hydrolysis of the processed sample
